# Transcriptomic analysis of male diamondback moth antennae: Response to female semiochemicals and allyl isothiocyanate

**DOI:** 10.1371/journal.pone.0315397

**Published:** 2024-12-19

**Authors:** Yueqin Zheng, Qianxia Liu, Houjun Tian, Hui Wei

**Affiliations:** 1 Institute of Plant Protection, Fujian Academy of Agricultural Sciences, Fuzhou, China; 2 Fujian Key Laboratory for Monitoring and Integrated Management of Crop Pests, Fuzhou Scientific Observing and Experimental Station of Crop Pests of Ministry of Agriculture, Fuzhou, China; Zhejiang Academy of Agricultural Sciences, CHINA

## Abstract

Female semiochemicals and allyl isothiocyanate (AITC) attract moths, and the moths use odorant-degrading enzymes (ODEs) to break down the excess odor. By identifying antennae-specific ODEs, researchers have established the molecular foundation for odorant degradation and signal inactivation in insects. This enables further exploration of new pest control methods. Currently, the degradation of female semiochemicals and AITC has received limited attention, inspiring this study to identify target ODEs in diamondback moths through transcriptome analysis. Sequencing of antennae from male adults (MA) exposed to female adults (FA) and AITC yielded a substantial 54.18 Gb of clean data, revealing 2276 differentially expressed genes (DEGs) between the MA and MA-FA treatments, and 629 DEGs between MA and MA-AITC treatments. The analysis of MAs exposed to FAs and AITC identified 29 and 17 ODEs, respectively, mainly involving *aldehyde dehydrogenases* (ALDHs), *alcohol dehydrogenases* (ADs), *cytochrome P450s* (CYPs), and *UDP-glucuronosyltransferases* (UGTs). Pathway analysis revealed primary enrichment in glycolysis/gluconeogenesis and fatty acid degradation in female adult treatments. In contrast, AITC treatments showed major enrichment in pathways related to pentose and glucuronate interconversions, retinol metabolism, and ascorbate and aldarate metabolism. Additionally, qRT-PCR analysis validated the expression patterns of 10 ODE genes in response to these treatments, with varying results observed among the genes. These findings indicate significant changes in ODE expression levels, providing a molecular foundation for identifying potential targets for behavioral inhibitors.

## Introduction

The diamondback moth, also known as DMB, or *Plutella xylostella* L. (Lepidoptera: Plutellidae), is a major agricultural pest. It causes vital damage to brassicaceous vegetables, with annual global economic losses estimated at US$4–5 billion [1,2]. Monitoring, trapping, and mating disruption using semiochemicals are key elements of green pest control and are extensively applied in agricultural practices [3–6]. Semiochemicals are substances or mixtures emitted by an organism that trigger behavioral or physiological responses in individuals of the same or different species, primarily influencing an insect’s behavior with plants or other insects [7]. Host plant volatiles provide critical resources for insects, facilitating feeding, mating, oviposition, and evasion of natural predators. The integration of female semiochemicals and plant volatile constitutes an eco-friendly approach to the monitoring and management of various pest populations [3,7].

The potential applications of pheromones from female semiochemicals are particularly important as they can help reduce the mating rates of female moths by 55% to 75%, hence mitigating crop damage [8]. The main components of DMB’s sex pheromones are (Z)-11-hexadecenal (Z11-16: Ald) and (Z)-11-hexadecenyl acetate (Z11-16: OAc). Using a ratio of 1:10 of sex pheromones and their analogs, specifically (11Z)-hexadec-11-en-1-yl2,2,2-trifluorooate and (11Z)-hexadec-11-en-1-yl 2,2,3,3-pentafluoropropanoate, nearly completely disrupts DMB mating activity in the field [9]. Allyl isothiocyanate (AITC) is a naturally occurring, highly volatile compound found in *Brassicaceae* family plants. It influences insect behaviors such as feeding and egg-laying and attracts adult moths [10–12]. AITC was shown to effectively control the pest *Bradysia odoriphaga* in plants both in fields and greenhouses [5]. Furthermore, high concentrations of AITC exhibit anti-insect and anti-bacterial properties, highlighting its potential for agricultural applications [13,14].

Insect antennae are crucial for detecting and distinguishing various semiochemicals [15,16]. In general, odor-binding proteins (OBPs) facilitate the transport of odor molecules to olfactory receptor neurons (ORNs) located in the antennae, where interaction with olfactory receptors (ORs) generates electrical signals, that are subsequently transmitted through axons to the brain [16]. Ultimately, odor-degrading enzymes (ODEs) eliminate excess odors, thereby preparing ORNs for new stimuli [15,17]. Several studies have employed transcriptomic analyses to identify olfactory genes, including research on *Dendrolimus houi* and *Dendrolimus kikuchii* [18], *Galleria mellonella* [19], and *Peridroma saucia* [20]. However, these investigations have seldom focused on ODEs. ODEs are grouped based on the chain lengths and functional groups present in the target odorant molecules, including key categories like carboxylesterases (CCEs/CXEs), aldehyde oxidases (AOXs) and ALDHs, CYPs, and ADs, etc. [21]. Enzymes such as CCEs /CXEs [22–25] and AOXs [15,26] are associated with the breaking down of sexual signals and plant volatiles. When exposed to strong odors, ODEs act as rapid odor inactivators, suppressing signals and influencing insect behavior by breaking down semiochemicals.

This study identified odor-degrading enzymes in the male adult (MA) antennae of the DMB that are sensitive to female adult (FA) and AITC through transcriptome analysis. Differential gene analysis, along with Gene Ontology (GO) and Kyoto Encyclopedia of Genes and Genomes (KEGG) analysis, revealed changes in the expression levels of *ALDHs*, *ADs*, *CYPs*, and *UGTs*. Our findings highlight specific ODEs that provide molecular target for the development of behavioral inhibitors aimed at utilizing female semiochemicals and AITC.

## Materials and methods

### Insect rearing

Diamondback moths were obtained from a cabbage (*Brassica oleracea* var. *capitata* L.) field without using an insecticide disposal in Jiyuan City, He nan province, China (112°60′E, 35°05′N). They were provided with feed and raised in a room at 26 ± 1°C,65 ± 5% humidity, and a 14L: 10 D photoperiod at the Plant Protection Institute of the Fujian Academy of Agricultural Sciences. After about 10 days of growth in the box from the egg stage, the 4^th^ instars were separated by gender [27] and each pupa was placed in a 2 mL glass tube with cotton plugging. Once they emerged, the adults received a 10% (w/v) honey solution.

### Insect collection

Male adult antennae were collected from the control group four days post-emergence. For the female adult treatment, male and female adults were housed in a 1:1 ratio in a box with double-layer gauze and male antennae were collected after 24 h. In the allyl isothiocyanate (AITC, Sigma-Aldrich Shanghai Trading Co., Ltd, W203408) treatment group, 0.1 μg/μL of AITC was applied to a 20 μL filter paper strip in the box, and male antennae were collected after 6 h. The dissecting table was disinfected with RnaseZap (Invitrogen, AM9780). Male adults were treated with CO_2_, immersed in 70% ethanol, and then dissected for antennae collection using forceps under a stereomicroscope. The antennae were immediately frozen in liquid nitrogen at -80°C. A total of 50 pairs of male adult antennae were used in both control and treatment groups, with each treatment consisting of three biological replicates.

### RNA extraction and sequencing

Total RNA was extracted from the male adult antennae using the TRIzol reagent (Invitrogen, 15596026CN) as per the manufacturer’s instructions. RNA concentration and purity were assessed with a Nanodrop2000 (Thermo Scientific), and RNA integrity was evaluated with an Agilent Bioanalyzer 2100 (Agilent Technologies). Sequencing libraries were prepared according to NEB (USA) guidelines, utilizing the NEBNext® Ultra™ RNA Library Prep Kit. Each sample received specific index codes for sequence attribution. The RNA-seq libraries underwent cluster generation with the TruSeq PE Cluster Kit v3-cBot-HS and the cBot Cluster Generation System from Illumina. Following library qualification, sequencing was conducted on the Illumina NovaSeq6000 platform in the PE150 mode, with each sample sequenced to a depth of 6 G. The unigene library for this species was generated through the assembly of clean data.

### Quality control

To ensure raw read suitability for analysis, specific quality measures were implemented. Reads containing adapters or showing low quality were removed. Specifically, reads with an N ratio over 10% and those with more than 50% of bases at a quality score of Q ≤ 10 were discarded. An evaluation was made of the Q20, Q30, GC-content, and sequence duplication levels of the clean data. The high-quality clean data were then provided in FASTQ format.

### Expression statistics and functional annotation

Reads from sequencing were compared using Bowtie [28], and expression levels were estimated with RSEM [29]. The expression abundance of each unigene was represented by the fragment number per kilobase transcript (FPKM) value [30]. Correlation coefficients between transcript and gene expressions were calculated with DESeq2 software [31]. This analysis identified differentially expressed genes (DEGs) between the comparison groups. DEGs were defined with criteria Fold Change≥2 and a false discovery rate (FDR) of less than 0.01 [32]. Principal Component Analysis (PCA) was utilized to moderate the dimensionality of gene expression datasets, thereby facilitating the examination of sample distribution patterns and the assessment of sample dispersion [33]. Moreover, to assess the reproducibility of biological experimental procedures, the reliability of DEGs, and identify any outlier samples, the relationship between gene expression levels across various samples was evaluated through correlation analyses [34]. Functional enrichment analysis was conducted using topGO [35] for GO analysis and KOBAS [36] for KEGG analysis, offering insights into the DEGs’ linked biological processes and pathways.

### Quantitative real-time PCR analysis and data analysis

Dissimilarities in gene expressions were determined by quantitative real-time polymerase chain reaction (qRT-PCR) following the method of Zheng et al. [37]. cDNA was synthesized from 1 μg of RNA using the HiScript® II 1st Strand cDNA Synthesis Kit (Vazyme). qPCR was performed with the Taq Pro Universal SYBR qPCR Master Mix (Vazyme) using *Pxylβ-tubulin* (GenBank accession number: *XM_011550528*.*1*) as an internal control. The primer sequences are provided in S3 Table and Table 3. The reaction comprised 5 μl of the 2×Taq Pro Universal SYBR qPCR Master Mix, 0.3 μl (0.3 μM) of each specific primer, 1 μl of cDNA, and the volume was adjusted to 10 μl with RNase-free water. Thermal cycling conditions included an initial denaturation at 95°C for 2 min, followed by 40 cycles of 95°C for 15 s and 60–64°C for 30 s. Amplicon specificity was confirmed through melting curve analysis. For qRT-PCR, three biological replicates were conducted and gene expression changes were measured using the 2^−ΔΔCt^ method with normalization [38]. Statistical analyses were performed using IBM SPSS Statistics version 21 (SPSS, Chicago, IL, USA), employing Student’s t-tests to compare treatment means.

## Results

### Transcriptome assembly and subsequent annotation

Our study analyzed transcriptomes from three groups: female adult treatment, allyl isothiocyanate treatment, and a control group of male adults. The primary aim was to assess the effects of exposure to female semiochemicals and plant-derived volatile compounds on gene expression. Transcriptome sequencing for all nine samples generated 54.18 Gb of clean data. Each sample produced at least 5.70 Gb, with a Q30 base percentage exceeding 91.75%, and an average GC content of 46.4% (Table 1). All clean reads are available in the NCBI database. The distribution of contig lengths, summarized in Table 2, shows 43,405 unigenes with an average length of 983.17 and an N50 of 1875. Notably, 50.73% of the contigs ranged from 200 to 500 bp, while 49.27% exceeded 500 bp, indicating strong assembly accuracy.

**Table 1:** Quality statistics of reads after raw filtering of *P*. *xylostella* transcriptomes from male antennas.

Sample Names	Read Number	Base Number	GC Content (%)	%≥Q30(%)
**MA-1**	20,336,664	6,077,635,772	46.64	94.36
**MA-2**	20,007,849	5,969,812,230	46.50	94.28
**MA-3**	19,807,348	5,920,106,086	46.34	94.26
**MA-FA-1**	20,081,716	6,001,452,576	46.99	94.11
**MA-FA-2**	20,713,808	6,184,639,190	46.80	94.05
**MA-FA-3**	19,099,355	5,706,083,310	46.92	94.40
**MA-AITC-1**	21,360,009	6,378,744,478	45.94	94.20
**MA-AITC-2**	20,796,990	6,211,483,148	45.52	94.21
**MA-AITC-3**	19,179,139	5,727,957,678	45.93	91.75

Read Number is the total number of pair-end reads in the clean data. MA, control; MA-FA, female exposed; MA-AITC, AITC exposed.

**Table 2:** Statistics of contigs assembled of *P*. *xylostella* transcriptomes from male antennas.

Length Range(nt)	Transcripts	Unigenes
**200–300**	15,438(20.76%)	13,169(30.34%)
**300–500**	12,837(17.26%)	8,852(20.39%)
**500–1000**	15,165(20.39%)	8,536(19.67%)
**1000–2000**	15,674(21.07%)	6,864(15.81%)
**2000+**	15,260(20.52%)	5,984(13.79%)
**Total Number**	74,374	43,405
**Total Length**	93,290,340	42,674,647
**N50 Length**	2,215	1,875
**Mean Length**	1254.34	983.17

The unigene N50 length denotes the length of the fragment at which the cumulative length reaches 50% of the total length of all unigenes.

### Enrichment analysis of DEGs

**Fig 1:**
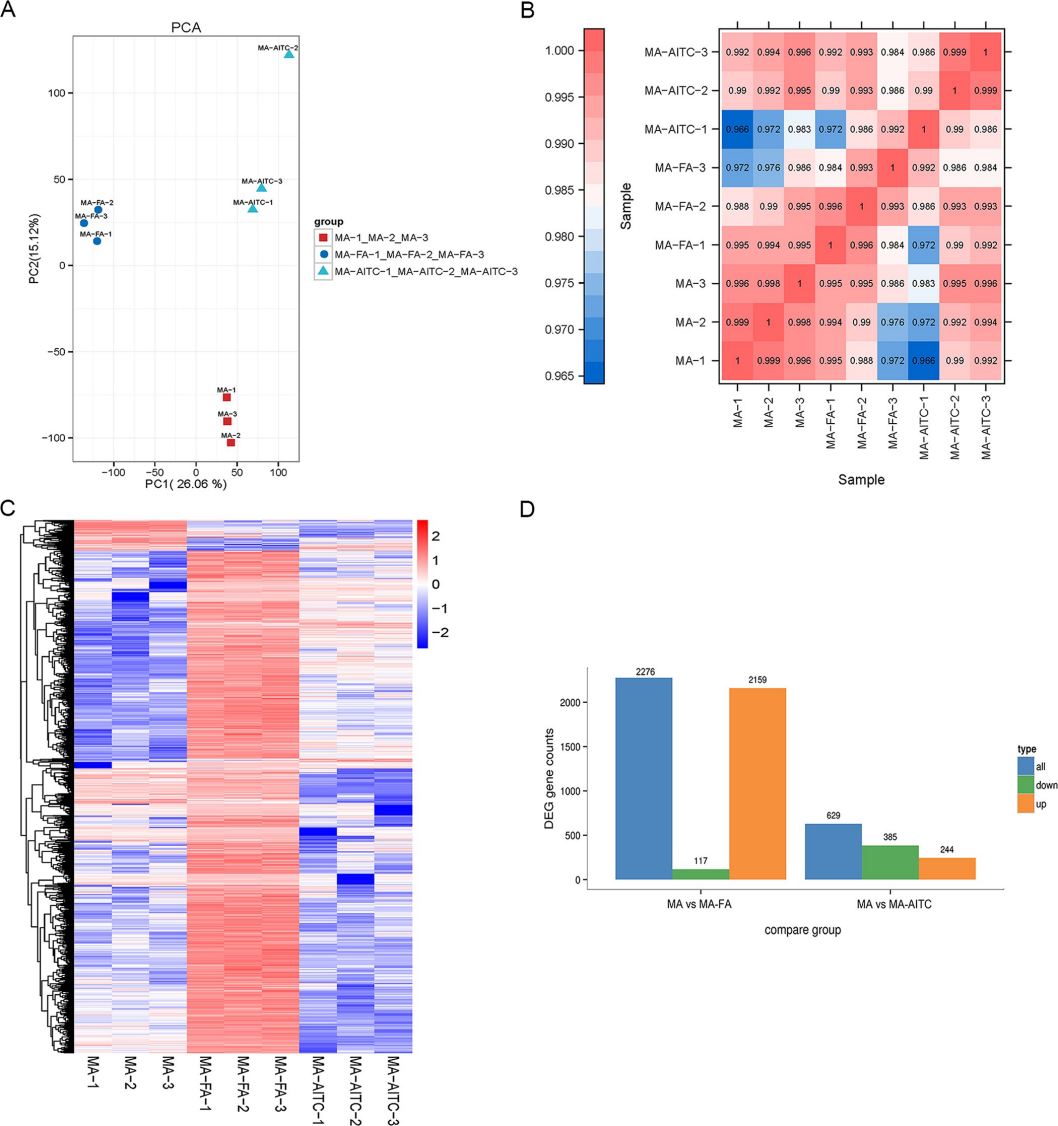
Transcriptome quality validation and differential gene analysis of *P*. *xylostella* male antennas (MA). Quality control metrics for transcriptomes are shown as principal components analysis (PCA) (A), correlation analysis (B), and gene cluster heatmap (C) for the MA, MA-FA, and MA-AITC groups. An overview of differentially expressed genes under different treatments (D). MA, control; MA-FA, female exposed; MA-AITC, AITC exposed.

Quality control of RNA-Seq data was assessed using PCA and correlation analyses (Fig 1A and 1B). The MA, MA-FA, and MA-AITC groups were well separated indicating notable variations in their expression levels (Fig 1A). Meanwhile, within the groups, a strong correlation in the levels of gene expression was identified (Fig 1B). Hierarchical clustering detected genes with similar expression patterns across samples, displayed as heatmaps. The comparison revealed a higher percentage of upregulated genes in the MA-FA group and more downregulated genes in the MA-AITC group (Fig 1C). Specifically, 2276 DEGs were identified between the MA and MA-FA treatments, and 629 DEGs between the MA and MA-AITC treatments (Fig 1D). Post-FA treatment, the number of genes upregulated in MA increased to 2159.

### Gene functional annotations of DEGs

**Fig 2:**
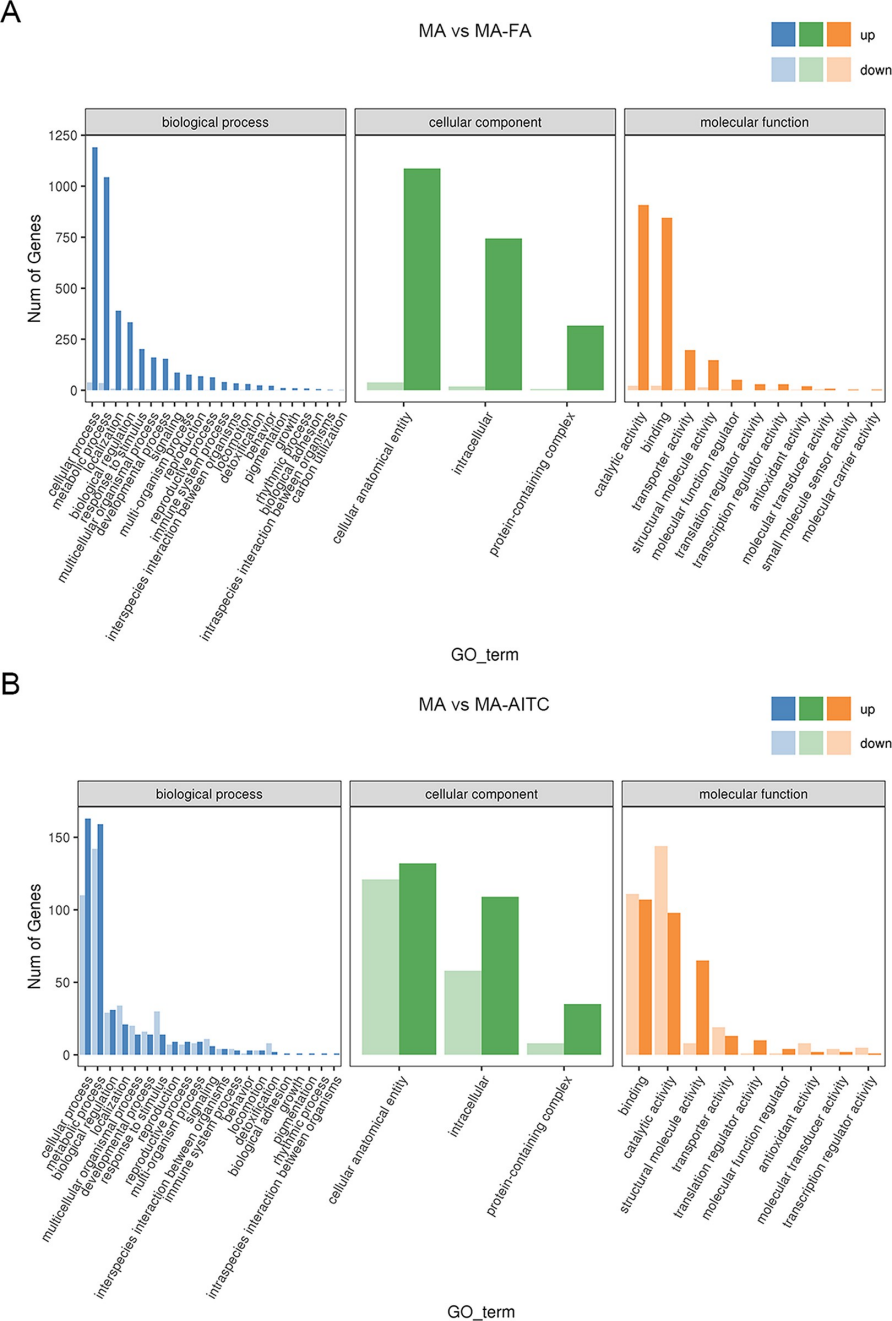
Gene Ontology (GO) enrichment of differentially expressed genes in MA vs MA-FA (A) and MA vs MA-AITC (B). The X-axis represents the GO classification, while the Y-axis represents the number of genes; colors indicate different primary classifications.

**Fig 3:**
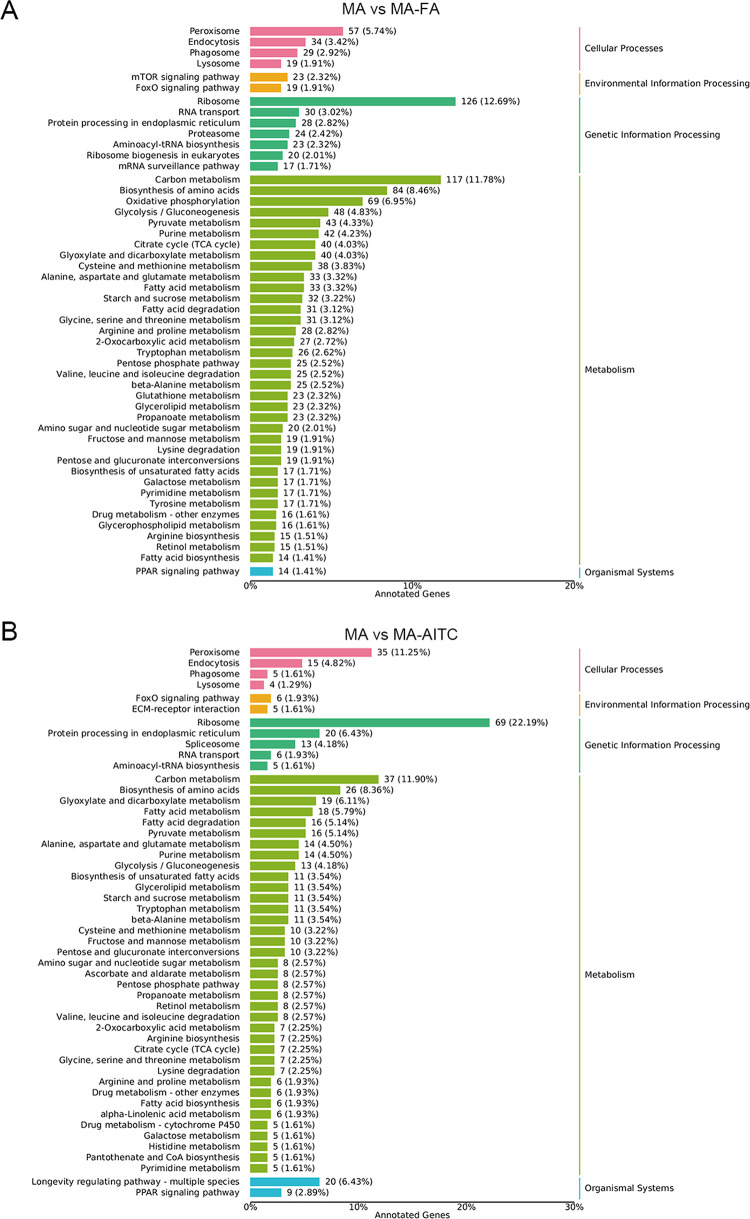
Kyoto Encyclopedia of Genes and Genomes (KEGG) analysis of differentially expressed genes in MA vs MA-FA (A) and MA vs MA-AITC (B). The left axis shows the annotated KEGG metabolic pathway names and the right axis shows the corresponding primary classification names. The X-axis represents the number of genes from the annotated pathway and their proportion to the total genes annotated.

The GO annotation analysis showed that the MA-FA treatment affected 1785 genes, with 1726 upregulated and 59 downregulated genes. In contrast, the MA-AITC treatment influenced 455 genes, with 210 upregulated and 245 downregulated genes. In biological processes, the cellular process pathway saw the most upregulated genes, with 1191 from the MA-FA treatment and 163 from the MA-AITC treatment. The metabolic process pathway also had significant upregulation, with 1044 and 159 genes from the MA-FA and MA-AITC treatments, respectively. Additionally, the cellular components analysis indicated 1087 upregulated genes in the cellular anatomical entity category from the MA-FA treatment and 132 from the MA-AITC treatment. In the molecular function category, the catalytic activity pathway had the highest upregulation, with 908 and 98 genes from the MA-FA and MA-AITC treatments, respectively. Following this, the binding pathway had 846 upregulated genes from the MA-FA treatment and 107 from the MA-AITC treatment (Fig 2A and 2B). The KEGG pathway analysis showed both treatments had enriched pathways like the ribosome, carbon metabolism, and amino acid biosynthesis (Fig 3A and 3B). The key difference was that MA-FA treatment enriched pathways were oxidative phosphorylation, mTOR signaling, and Proteasome (Fig 3A), while the MA-AITC treatment pathways were specific to longevity regulation and spliceosome (Fig 3B).

### Changes in the expression of ODEs

**Fig 4:**
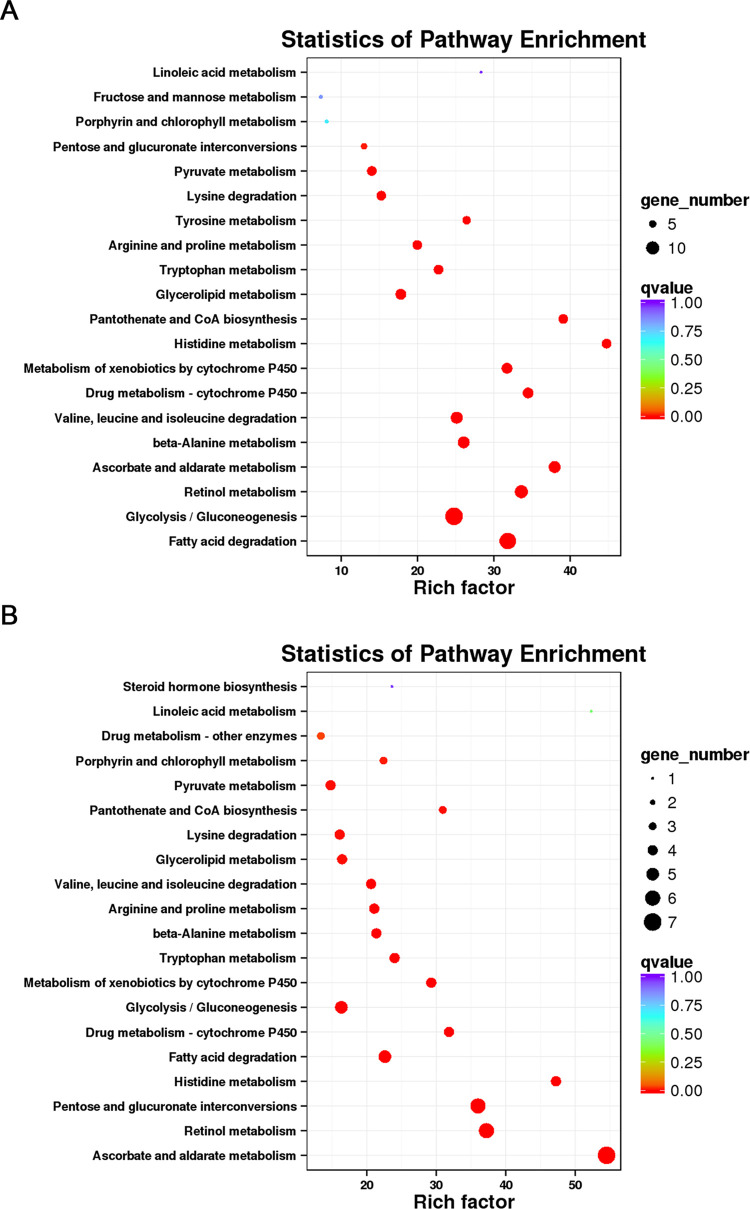
KEGG enrichment analysis of odorant-degrading enzymes in MA vs MA-FA (A) and MA vs MA-AITC (B). The enriched pathways were visualized with q-values (marked in represented colors); the enrichment factor is on the X-axis, and the number of involved differentially expressed genes is indicated by the corresponding circle size.

**Fig 5:**
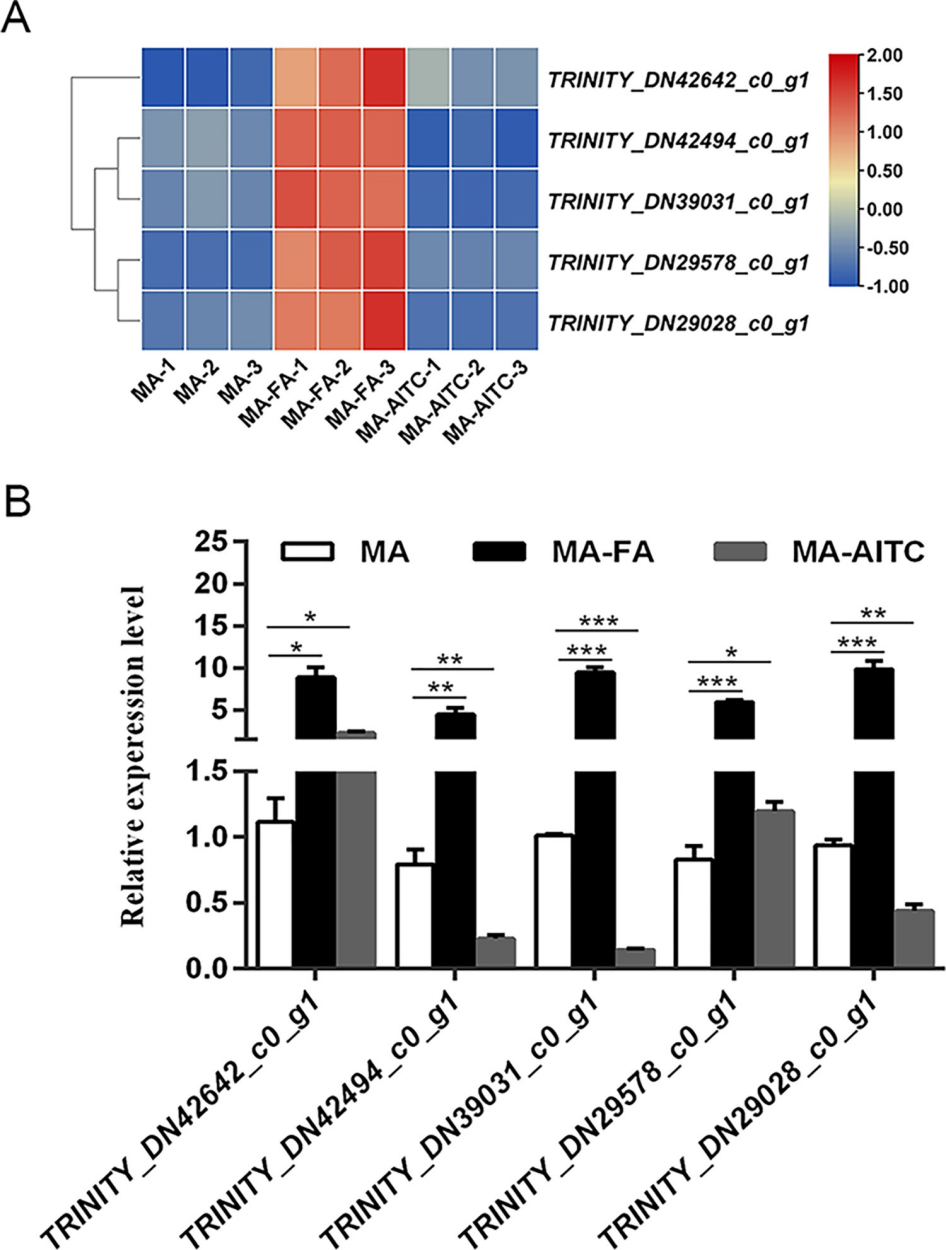
Transcriptomic (A) and qRT-PCR (B) expression levels of five odorant-degrading enzyme genes were compared between control (MA) and female exposed (MA-FA), as well as between control (MA) and AITC exposed (MA-AITC). Values are the mean ± SE (n = 3). Statistical significance was determined by Student’s t-test: * P < 0.05, ** P < 0.01, or *** P < 0.001.

**Fig 6:**
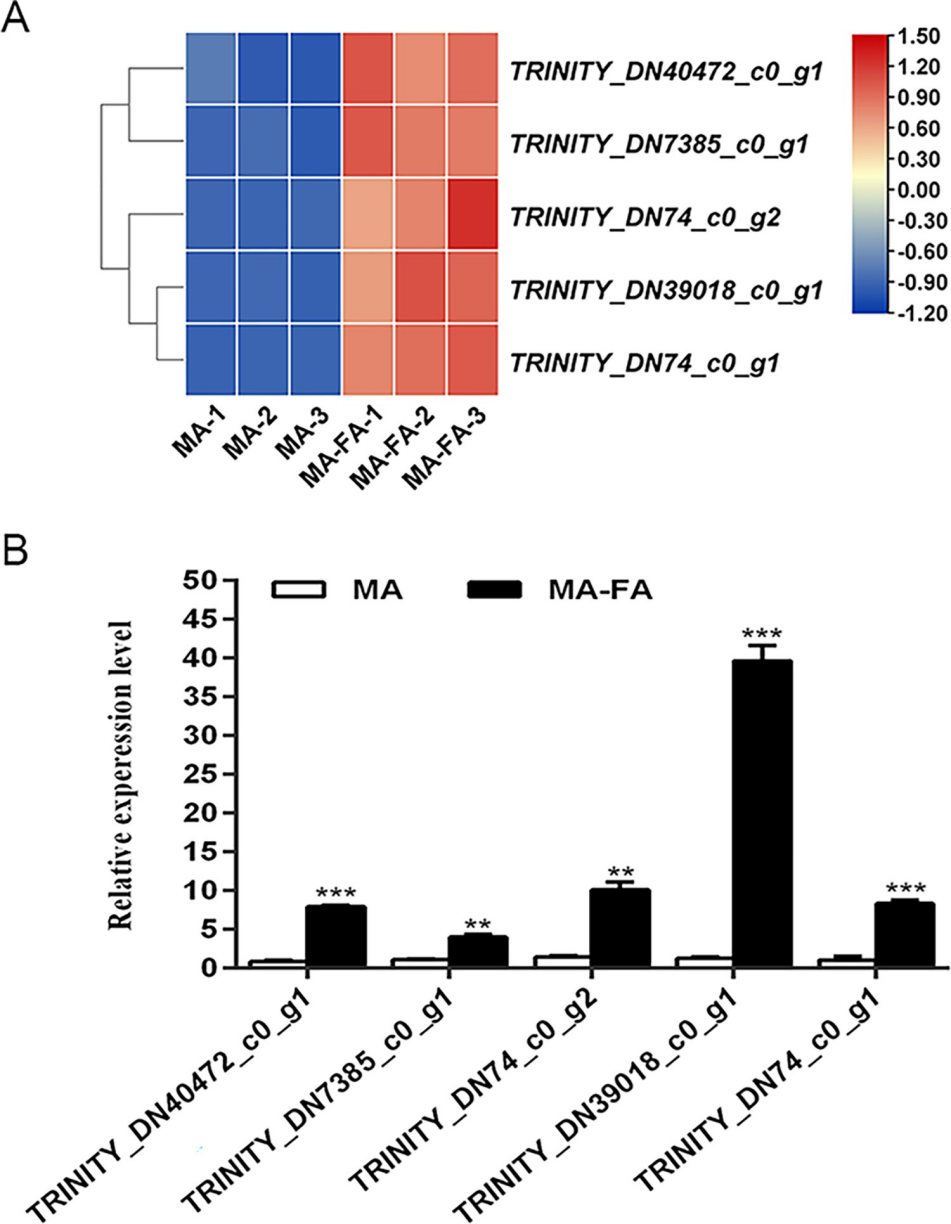
Transcriptomic (A) and qRT-PCR (B) expression levels of five odorant-degrading enzyme genes were compared between control (MA) and female exposed (MA-FA). Values are the mean ± SE (n = 3). Statistical significance was determined by Student’s t-test: ** P < 0.01, or *** P < 0.001.

This study found differential expression of 29 ODE genes in the MA vs MA-FA comparison (S1 Table) and 17 in the MA vs MA-AITC comparison (S2 Table). The MA vs MA-FA group showed primary pathway enrichment in glycolysis/gluconeogenesis and fatty acid degradation (Fig 4A), while the MA vs MA-AITC group was major enriched in pentose and glucuronate interconversions, retinol metabolism, and ascorbate and aldarate metabolism pathways (Fig 4B). Comparison among MA, MA-FA, and MA-AITC groups identified five genes with higher expression for further investigation. *TRINITY_DN42642_c0_g1* and *TRINITY_DN29578_c0_g1* were upregulated in both MA-FA and MA-AITC groups compared to MA. The remaining three genes were downregulated in the MA-AITC group but significantly upregulated in the MA-FA group (Table 3 and Fig 5). Five genes were notably upregulated in the MA-FA group, with *TRINITY_DN39018_c0_g1* showing a significant 30.0-fold upregulation in the MA-FA group compared to MA (Table 3 and Fig 6). Additionally, a decrease in the expression of five heat shock protein (Hsp) genes was noted in the MA-AITC group compared to MA (S3 Table and S1 Fig). The qRT-PCR analysis validated the reliability of the DEG data by confirming the RNA-seq data.

**Table 3:** qRT-PCR primers used for odor degradation enzyme in *P*. *xylostella* male antenna.

Gene Name	Gene ID	Sequences (5’-3’)
***Tubulin***	*XM_011550528*.*1*	CAATCAGGCCAATTTACCGC CTGGGTTTACGCCAGTTACG
***ALDH***	*TRINITY_DN29578_c0_g1*	GCCAAACGGGACCTCTTAT GGCAACAGCACGGTCAAT
***ALDH***	*TRINITY_DN42494_c0_g1*	AGGTTGCCTTCACTGGCTCTA CCACTTGGCGGCTTGTT
***CYP***	*TRINITY_DN42642_c0_g1*	AGAGCGAAGAGGAAGGTGAAA TGCTTGCGTTATGGATGAATG
***CYP***	*TRINITY_DN39031_c0_g1*	AGATGGTGCTGGGTGGAA CCTTGTGCTGGCGGATAT
***AD***	*TRINITY_DN29028_c0_g1*	CGGCCATCAATTCGTAAACC GATTCCGTTGTCAAGACCATTTC
***ALDH***	*TRINITY_DN40472_c0_g1*	GTCTGATTTGGGACCACTCG CCCTTAGCGAACTTCCCTTT
***ALDH***	*TRINITY_DN39018_c0_g1*	AGAATCCCTTGTCTCCGTGTC CACCTTCATGGGTGCTCAA
***CYP***	*TRINITY_DN7385_c0_g1*	GACCCTCGATCCACCTTCAC TGTGCTTAGGCAGGATATAGGC
***AD***	*TRINITY_DN74_c0_g1*	CCAGTTGGGCGAACGTAGTCA TGGTTTGGGAACTCTTGCTGTG
***AD***	*TRINITY_DN74_c0_g2*	GCAGCCTGGACAGCATCA TTGGCCGGTATCAAGTGGT

## Discussion

Past research has mostly concentrated on the screening and identification of olfactory proteins, such as OBPs, ORs, and chemosensory proteins (CSPs), while neglecting ODEs. In this study, we explored the response of male DBMs to the female semiochemicals and plant volatile compound AITC. We mainly aimed to screen for ODEs capable of degrading the female semiochemicals and AITC. By identifying ODEs that respond to specific chemical signals, this research offers insights into potential novel strategies for pest control.

Previous research has indicated that exposure to various odors alters the expression of ODEs in insect antennae [21,22,39]. In a transcriptome analysis of *Grapholita molesta*, 23 candidate CXEs were identified. Notably, *GmolCXE1* and *GmolCXE5* showed significant upregulation in the antennae of male moths in response to female adults, while *GmolCXE14* and *GmolCXE 21* displayed approximately two-fold upregulation in larval heads in response to fresh mature fruit odors. These genes may play a role in degrading sex pheromones or fruit odors, potentially affecting mating and foraging behaviors [22]. Additionally, research on DBM showed that two homologous carboxylesterases, *CCE16a* and *CCE16c*, can degrade sex pheromones and plant volatiles. CCE16c exhibited greater hydrolysis activity than CCE16a, indicating its potential role in odorant degradation [21]. Using exposure to female semiochemicals and AITC, we also screened several ODEs. In the MA vs MA-FA comparison, 29 ODEs were upregulated in male antennae (S1 Table) that are primarily enriched in glycolysis/glycogenesis and fatty acid degradation pathways (Fig 4A). This suggests that male insects may upregulate these genes to degrade excess female semiochemicals (S1 Table and Figs 5 and 6). After exposure to AITC, 17 ODEs were identified. These are majorly enriched in pentose and glucuronate interconversions, retinol metabolism, and ascorbate and aldarate metabolism pathways. The insect’s response to AITC is concentration-dependent, where moderate AITC levels are promoting [40] while high levels are toxic [41,42]. During a 6 h AITC exposure, 14 ODE genes were downregulated and 3 were upregulated (S2 Table and Fig 5), indicating a subtle response based on treatment duration and concentration. Notably, in our preliminary experiments, known ODE expressions exhibited an increase following a 6 h treatment with AITC. This initial finding guided our decision to focus on the 6 h time point for subsequent transcriptomic analysis. Nevertheless, the findings from the detailed study indicate that 6 h treatment with AITC may not be the optimal time for identifying the upregulated ODE expressions effectively.

Furthermore, we identified four classes of ODEs (ALDHs, ADs, CYPs, and UGTs), particularly *ALDHs* and *CYPs* showing the most significant expression changes. Previous studies demonstrated that *ALDHs* metabolize volatile compounds such as acetaldehyde [43], ethanol [44], farnesal [45], and verbenone [46]. Insect cytochrome P450 monooxygenases are involved in metabolizing and detoxifying external substances, including plant chemicals, insecticides, and environmental pollutants [47–49]. Knockout experiments targeting the CYP6AE subfamily have shown increased sensitivity to insecticides and plant chemicals, highlighting the importance of *CYPs* in insect physiology [50,51]. This suggests that these target genes hold potential for further research.

In this study, we identified ODEs associated with the degradation of female semiochemicals and AITC through transcriptome screening. These can be categorized into four groups: ALDHs, ADs, CYPs, and UGTs. Future studies should prioritize genes with increased *ALDHs* and *CYPs* expression levels. To facilitate this, we should conduct bioinformatics assessments, cloning, prokaryotic expression, and protein assays to evaluate the degradation of female semiochemicals and AITC in DBM. Given the fact that even minimal quantities of volatiles can be lethal to insects that are incapable of decomposing scents, the application of ds*ODEs* in the use of semiochemicals can markedly lower the cost of pest control.

## Supporting information

S1 Fig Transcriptomic (A) and qRT-PCR (B) expression levels of five heat shock protein genes were compared between control (MA) and AITC exposed (MA-AITC). Values are the mean ± SE (n = 3). Statistical significance was determined by Student’s t-test: ** P < 0.01, or *** P < 0.001.
(DOCX)

S1 Table Gene expressions of odorant-degrading enzymes in the antennae transcriptome of male diamondback moths in control and female exposed. (DOCX)

S2 Table Gene expressions of odorant-degrading enzymes in the antennae transcriptome of male diamondback moths in control and AITC exposed. (DOCX)

S3 Table Primers of heat shock protein genes in *P*. *xylostella* male antennas used for real-time qRT-PCR in control and AITC exposed. (DOCX)
