# Supplementary material for: Transcriptomic analysis of male diamondback moth antennae: Response to female semiochemicals and allyl isothiocyanate
Source: PLoS One. 2024 Dec 19;19(12):e0315397. doi: 10.1371/journal.pone.0315397 (PMC11658498; doi:10.1371/journal.pone.0315397)
Supplement: S1 Fig — Transcriptomic (A) and qRT-PCR (B) expression levels of five heat shock protein genes were compared between control (MA) and AITC exposed (MA-AITC). Values are the mean ± SE (n = 3). Statistical significance was determined by Student’s t-test: ** P < 0.01, or *** P < 0.001. (DOCX) [file pone.0315397.s001.docx]

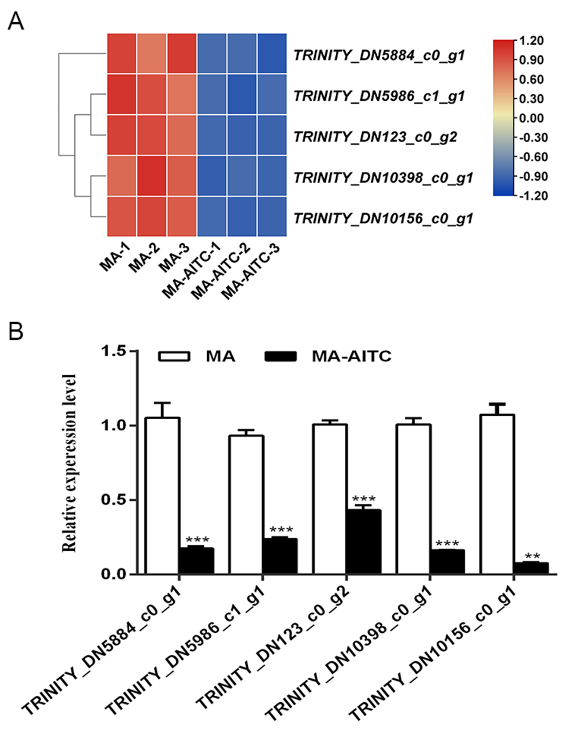


**S1 Fig. Transcriptomic (A) and qRT-PCR (B) expression levels of five heat shock protein genes were compared between control (MA) and** **AITC exposed (MA-AITC).** Values are the mean ± SE (n = 3). Statistical significance was determined by Student’s t-test: ** P < 0.01, or *** P < 0.001.
