# Supplementary material for: Transcriptomic analysis of male diamondback moth antennae: Response to female semiochemicals and allyl isothiocyanate
Source: PLoS One. 2024 Dec 19;19(12):e0315397. doi: 10.1371/journal.pone.0315397 (PMC11658498; doi:10.1371/journal.pone.0315397)
Supplement: S1 Table — (DOCX) [file pone.0315397.s002.docx]

**S1 Table. Gene expressions of** **odorant-degrading enzymes in the antennae transcriptome of male diamondback moths in control and female exposed.**

| **Gene** | **Gene ID** | **FDR** | **log_2_FC** |
| --- | --- | --- | --- |
| **Aldehyde dehydrogenase** | *TRINITY_DN5502_c0_g1* | 0.001537187 | 1.887100163 |
|  | *TRINITY_DN39018_c0_g1* | 1.56161E-93 | 3.45961016 |
|  | *TRINITY_DN17373_c0_g1* | 0.000130654 | 2.624432177 |
|  | *TRINITY_DN23052_c0_g1* | 5.13904E-07 | 1.866897091 |
|  | *TRINITY_DN41495_c0_g1* | 1.64141E-06 | 2.343708806 |
|  | *TRINITY_DN42494_c0_g1* | 9.44998E-15 | 1.934305688 |
|  | *TRINITY_DN19812_c2_g1* | 2.55351E-18 | 1.841070291 |
|  | *TRINITY_DN29578_c0_g1* | 0 | 3.651923375 |
|  | *TRINITY_DN15264_c0_g1* | 1.23358E-06 | 3.677205101 |
|  | *TRINITY_DN40472_c0_g1* | 1.67736E-15 | 3.589020646 |
| **Cytochrome P450** | *TRINITY_DN13890_c0_g1* | 8.51434E-11 | 3.619599122 |
|  | *TRINITY_DN42642_c0_g1* | 5.65238E-16 | 3.230596418 |
|  | *TRINITY_DN39031_c0_g1* | 1.71418E-47 | 2.570047703 |
|  | *TRINITY_DN20125_c0_g1* | 4.34875E-18 | 2.132526047 |
|  | *TRINITY_DN7385_c0_g1* | 4.78532E-59 | 1.524122049 |
|  | *TRINITY_DN8676_c1_g1* | 2.96407E-05 | 1.479761669 |
|  | *TRINITY_DN3874_c0_g1* | 1.82545E-21 | 1.300833277 |
|  | *TRINITY_DN37067_c0_g1* | 1.45286E-08 | 1.283767815 |
| **Alcohol dehydrogenase** | *TRINITY_DN43780_c0_g1* | 7.01848E-22 | 1.872419747 |
|  | *TRINITY_DN29028_c0_g1* | 1.58897E-38 | 2.84074231 |
|  | *TRINITY_DN42067_c0_g1* | 0.004243437 | 1.910341991 |
|  | *TRINITY_DN44487_c0_g1* | 0.001058025 | 1.656659114 |
|  | *TRINITY_DN74_c0_g1* | 0 | 3.883027654 |
|  | *TRINITY_DN15272_c0_g1* | 1.89798E-16 | 2.455408729 |
|  | *TRINITY_DN42919_c0_g1* | 4.03094E-16 | 2.880313409 |
|  | *TRINITY_DN74_c0_g2* | 9.90721E-75 | 3.037278853 |
|  | *TRINITY_DN39269_c0_g1* | 3.33208E-05 | 3.006026887 |
| **UDP-glucosyl transferase** | *TRINITY_DN37233_c0_g1* | 1.2465E-20 | 1.463800728 |
|  | *TRINITY_DN2583_c0_g1* | 2.29216E-62 | 1.372518887 |
