# Supplementary material for: Transcriptomic analysis of male diamondback moth antennae: Response to female semiochemicals and allyl isothiocyanate
Source: PLoS One. 2024 Dec 19;19(12):e0315397. doi: 10.1371/journal.pone.0315397 (PMC11658498; doi:10.1371/journal.pone.0315397)
Supplement: S2 Table — (DOCX) [file pone.0315397.s003.docx]

**S2 Table.** **Gene expressions of odorant-degrading enzymes in the** **antennae transcriptome of male diamondback moths** **in control and AITC exposed.**

| **Gene** | **Gene ID** | **FDR** | **log_2_FC** |
| --- | --- | --- | --- |
| **Aldehyde dehydrogenase** | *TRINITY_DN5228_c0_g1* | 2.38E-06 | -1.312276944 |
|  | *TRINITY_DN42494_c0_g1* | 2.87E-05 | -2.288051658 |
|  | *TRINITY_DN19812_c2_g1* | 5.18E-09 | -2.292056823 |
|  | *TRINITY_DN29578_c0_g1* | 1.90E-23 | 1.077185293 |
| **Cytochrome P450** | *TRINITY_DN8676_c1_g1* | 9.25437E-05 | -3.271938512 |
|  | *TRINITY_DN6071_c1_g2* | 0.000469969 | -1.101934985 |
|  | *TRINITY_DN39031_c0_g1* | 7.73168E-14 | -3.496243707 |
|  | *TRINITY_DN42642_c0_g1* | 0.005721451 | 1.617047619 |
|  | *TRINITY_DN20125_c0_g1* | 0.001721532 | -1.574619613 |
|  | *TRINITY_DN37067_c0_g1* | 7.28051E-17 | -3.835847596 |
| **Alcohol dehydrogenase** | *TRINITY_DN43780_c0_g1* | 0.004321067 | -1.005296131 |
|  | *TRINITY_DN29028_c0_g1* | 0.007205375 | -1.23102647 |
|  | *TRINITY_DN2120_c0_g1* | 1.0452E-10 | -1.281613421 |
|  | *TRINITY_DN4033_c0_g1* | 5.57678E-09 | -1.122095523 |
| **UDP-glucosyl transferase** | *TRINITY_DN43822_c0_g1* | 0.001080209 | -1.195422724 |
|  | *TRINITY_DN39273_c0_g1* | 0.000687748 | 1.488907087 |
|  | *TRINITY_DN6972_c0_g1* | 5.30272E-16 | -1.027883454 |
