# Supplementary material for: Transcriptomic analysis of male diamondback moth antennae: Response to female semiochemicals and allyl isothiocyanate
Source: PLoS One. 2024 Dec 19;19(12):e0315397. doi: 10.1371/journal.pone.0315397 (PMC11658498; doi:10.1371/journal.pone.0315397)
Supplement: S3 Table — (DOCX) [file pone.0315397.s004.docx]

**S3 Table. Primers of** **heat shock protein genes in *P. xylostella* male antennas used for real-time qRT-PCR in control and AITC exposed.**

| **Gene** | **Gene ID** | **Sequence (5’-3’)** |
| --- | --- | --- |
| **Heat shock proteins** | *TRINITY_DN5986_c1_g1* | ACCGACGCTTGAACGACCTG  CCTCCCTGACGCTACCAGACAT |
|  | *TRINITY_DN10398_c0_g1* | GGAAGGAAATCAAGGACCAA  ATGCCAATGCAGTTACACTCAC |
|  | *TRINITY_DN5884_c0_g1* | ACCAGCCCGCCGTTACCAT  CCCGTTAGCGTCCATGTCGA |
|  | *TRINITY_DN123_c0_g2* | GCCTTCACCTCGAACAGCG  GCATCATCAACGAGCCCACC |
|  | *TRINITY_DN10156_c0_g1* | TACGCTTCGGCGGTCTCCTT  GACTGCGGCAAGCCCAAGAT |
